# Pruritus Is an Indicator for Quality of Life in Cutaneous T‐Cell Lymphoma

**DOI:** 10.1111/1346-8138.17847

**Published:** 2025-07-18

**Authors:** Andrea Roggo, Yves Wüest, Reinhard Dummer, Egle Ramelyte

**Affiliations:** ^1^ Department of Dermatology University Hospital of Zurich, University of Zurich Zurich Switzerland; ^2^ Department of Dermatology Kantonsspital Aarau Aarau Switzerland

**Keywords:** cutaneous T‐cell lymphoma, pruritus, quality of life, questionnaire

## Abstract

In cutaneous T‐cell lymphoma (CTCL), chronic pruritus affects up to 94% of patients as a leading factor reducing quality of life (QoL). Traditional antipruritic medication does not show sufficient relief. Partly due to a lack of therapeutic options, pruritus is rarely assessed in a standardized manner within routine clinical practice. In this cross‐sectional, single‐institution study, we characterized CTCL‐associated pruritus including perception, dynamics, and modifying factors. QoL was evaluated with the Dermatology Life Quality Index (DLQI) and EORTC core quality of life questionnaire (QLQ‐C30). Patients with early‐stage Mycosis fungoides (MF, *n* = 16), late‐stage MF (*n* = 10) and Sézary syndrome (SS, *n* = 10), treated at the University Hospital of Zurich, completed all three questionnaires. 80.5% (*n* = 29/36) patients reported disease‐associated pruritus worsening with stress (51.7%, *n* = 15/29), scratching (37.9%, *n* = 11/29), and sweating or exercise (31.0%, *n* = 9/29). DLQI was associated with CTCL‐specific skin involvement (modified severity‐weighted assessment tool, mSWAT) (ρ = 0.44, *p* < 0.01), worst experienced pruritus (*ρ* = 0.42, *p* = 0.04) and current perceived pruritus (*ρ* = 0.60, *p* < 0.01). Summarizing those three factors in a Pruritus‐Score correlated strongly with DLQI (*ρ* = 0.71, *p* < 0.01), QLQ‐C30 global health status (*ρ* = −0.71, *p* < 0.01) as well as fatigue (*ρ* = 0.50, *p* = 0.02). Our results confirm the relevance of pruritus in CTCL. The Pruritus‐Score outperforms solely assessment of present pruritus in identifying patients who might experience related impairments in QoL. It quantifies this complex symptom in three factors that can be collected in clinical practice. This Pruritus‐Score can support consideration of pharmaceutical and non‐pharmaceutical approaches aimed at addressing reductions in quality of life and associated symptoms such as fatigue.

## 1 Introduction

Cutaneous T‐cell lymphoma (CTCL) is originating from malignant T‐cells accumulated in the skin [1]. Mycosis fungoides (MF) and Sézary syndrome (SS) are subtypes with localized and systemic manifestation, respectively [1]. MF, the most common type of CTCL, presents with plaques or patches that might be accompanied by alopecia in the folliculotropic subtype [1]. Early‐stage MF with minor skin involvement is distinguished from late‐stage MF, where skin lesions are pronounced and prognosis is worse [2]. SS, as the most aggressive, systemic type of CTCL, occurs with erythroderma and generalized lymphadenopathy [1]. Cutaneous involvement of CTCL is assessed and monitored with the modified severity‐weighted assessment tool (mSWAT) [3]. In clinical practice, pruritus severity can be scored with a visual analogue scale (VAS) or numeric rating scale (NRS) [4]. Up to 94% of CTCL patients report intractable, chronic pruritus rarely responding to antipruritic therapy [4].

In advanced CTCL, health‐related quality of life (QoL) including functional, emotional, and physical domains might be impaired in patients independent of the ongoing treatment [5, 6, 7]. Pruritus is the most frequent and bothersome symptom reported [6]. While established QoL questionnaires such as the Dermatology Life Quality Index (DLQI) [8] and EORTC Core Quality of Life Questionnaire (QLQ‐C30) [9] are optimized for dermatological and cancer‐related symptoms, they are not tailored to the specific challenges of CTCL. For patients with chronic pruritus, ItchyQoL is superior in gaining a detailed understanding of the influence of pruritus [10, 11]. In CTCL, all domains of ItchyQoL such as symptoms, functioning, and emotions are impaired [12], underlying the need for a CTCL‐specific tool taking into account the disease characteristics and pruritus to assess QoL.

In this study, we perform a comprehensive characterization of pruritus, evaluating relevant domains such as frequency, severity, and characterization. Second, we assess the relevance of the individual items as indicators for patients QoL. On this basis, we build a short, applicable Pruritus‐Score to identify CTCL patients with impaired QoL and related symptoms.

## 2 Methods

This is a single‐institution, cross‐sectional study with both retrospective chart review and prospective data completion of three questionnaires.

### 2.1 Participant Recruitment

Patients diagnosed with MF or SS, treated from June to July 2023 at the Department of Dermatology, University Hospital of Zurich (USZ), were included in the study population. The institutional database for medical records was reviewed for the identification of eligible participants to complete a target size of at least 10 participants per subgroup (Stage IA‐IIA as early‐stage MF, Stage IIB‐IV as late‐stage MF, and SS) [13]. The sample size is limited due to the low incidence of SS. Additional inclusion criteria were age from 18 to 100 years and a fluent understanding of the written German language. Selected participants were contacted by phone or approached during the clinic appointment.

### 2.2 Assessment of QoL and Pruritus

Consenting patients received three questionnaires along with a cover letter and a prepaid return envelope. For assessment of QoL, the official German version of DLQI [8] and QLQ‐C30 [9] were selected. DLQI score ranges from 0 (no impairment) to 30 (maximum impairment). QLQ‐C30 scales symptom severity in a score from 0 (not present) to 100 (maximal impact). Global health status and function scales range from 0 (no function) to 100 (complete function).

Pruritus‐specific characteristics were evaluated with the CTCL Pruritus Questionnaire, designed in the German language based on established multiple‐choice pruritus questions [14] as well as added CTCL‐specific open questions including characteristics, specific antipruritic treatment, and relation to skin involvement. A diagram showing the human body was implemented to mark the involved body areas [15]. The questions were translated into the English language and structured into the following chapters: onset/course, localization, characterization, influencing factors, and antipruritic treatment (Figure S1).

### 2.3 Data Collection

Upon consent, demographic data (sex and age) and disease‐specific parameters (disease stage, histopathological subtype, current skin involvement, type and duration of topical and systemic treatment) were retrospectively collected from the medical records of the USZ.

### 2.4 Statistical Analysis

Analyses were done using open‐source statistic software R Studio (v 4.3.1) with packages readxl (v 1.4.2), tableone (v 1.13.2), tidyverse (v 2.0.0), psych (v 2.3.3) as well as Microsoft Excel 365 (v 2308). Statistical analysis includes Spearman's rho rank‐order correlation. Spearman's correlation coefficient (*ρ*) was interpreted as follows: *ρ* = 0–0.1, no correlation; *ρ* = 0.1–0.29, weak correlation; *ρ* = 0.3–0.49, moderate correlation; *ρ* = 0.5–0.7, strong correlation; *ρ* > 0.7, very strong correlation. Statistical significance was determined based on adjusted *p*‐values < 0.05, using the Benjamini‐Hochberg method to control the false discovery rate. Internal consistency of factors contributing to the Pruritus‐Score was assessed using Cronbach's alpha (*α*).

Missing data in the QLQ‐C30 questionnaire was extrapolated in accordance with the recommended scoring manual (3rd Version) [16]. There were no missing data in the DLQI. For the CTCL Pruritus Questionnaire, a scoring manual ordering qualitative results and imputation of missing data is included (Table S1). Half‐maximal value imputation was chosen to preserve sample size while minimizing distortion of variance and mean.

## 3 Results

### 3.1 Study Population

In this study, 36 patients diagnosed with early‐stage MF (44.4%, *n* = 16/36), late‐stage MF (27.8%, *n* = 10/36) and SS (27.8%, *n* = 10/36) were included (shown in Table 1). Mean age was 69.6 years (±10.0 years). The majority was male (58.3%). Cutaneous involvement at the time of data collection scored a mean mSWAT of 18.6 (±21.8), covering a spectrum of low to high skin involvement. Overall pruritus prevalence was 80.5% (*n* = 29/36) with 58.3% (*n* = 21/36) of all patients experiencing pruritus at the time of data collection. Reported antipruritic treatment included Bilastin (16.7%, *n* = 6/36), Levocetirizine (5.6%, *n* = 2/36) and Gabapentin (8.3%, *n* = 3/36).

**TABLE 1:** Patient characteristics.

Parameters	CTCL cohort (*n* = 36)
Stage, *n* (%)	
Early‐stage MF	16 (44.4)
Late‐stage MF	10 (27.8)
SS	10 (27.8)
Age, years, mean ± SD	69.6 ± 10.0
Sex, male, *n* (%)	21 (58.3)
mSWAT, mean ± SD	18.6 ± 21.8
Topical treatment, *n* (%)	
Steroid	34 (94.4)
nbUVB	3 (8.3)
PUVA	3 (8.3)
Radiotherapy	4 (11.1)
Systemic treatment, *n* (%)	
ECP	7 (19.4)
Methotrexate	7 (19.4)
Brentuximab	1 (2.8)
Mogamulizumab	3 (8.3)
Acitretin	1 (2.8)
Peginterferon α2a	4 (11.1)
Status post ASCT	1 (2.8)
Pruritus prevalence, *n* (%)	29 (80.5)
Pruritus incidence, *n* (%)	21 (58.3)
Antipruritic treatment, *n* (%)	
Bilastin	6 (16.7)
Levocetirizine	2 (5.6)
Gabapentin	3 (8.3)

Abbreviations: ASCT, autologous stem cell transpant; ECP, extracorporeal phrotopheresis; FMF, folliculotropic Mycosis fungoides; MF, Mycosis fungoides; mSWAT, modified severity‐weighted assessment tool; nbUVB, narrowband ultraviolet phototherapy; PUVA, psoralen and ultraviolet light therapy; SS, Sézary syndrome; CTCL, cutaneous T‐cell lymphoma; SD, standard deviation.

### 3.2 Pruritus Characteristics Are Heterogeneous in CTCL

**FIGURE 1:**
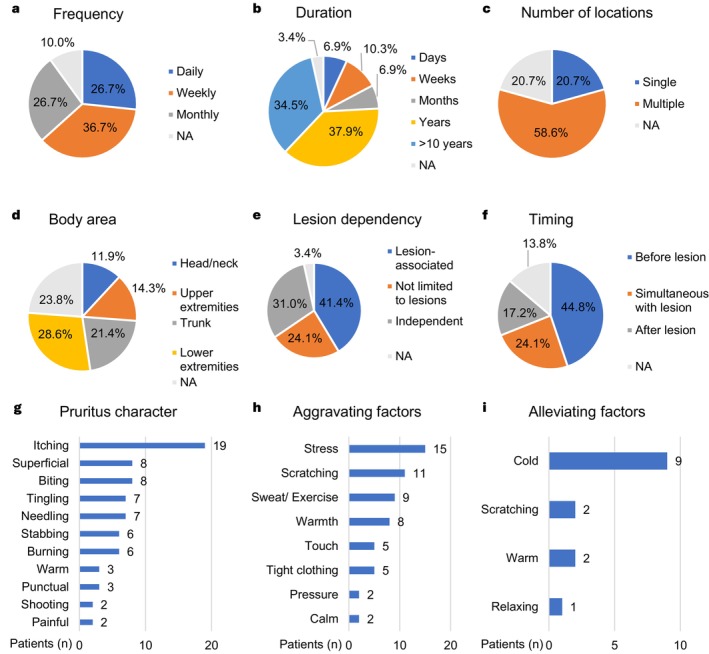
Pruritus characteristics in CTCL. Descriptive statistics of pruritus characteristics among CTCL patients reporting pruritus (*n* = 29). Subpanels (a‐f) display the distribution of key pruritic features using pie charts: (a) frequency of pruritus episodes (daily, weekly, monthly), (b) symptom duration (days, weeks, months, years, > 10 years), (c) number of pruritic lesions (single, multiple), (d) involved anatomical location (head/neck, upper extremities, trunk, lower extremities), (e) relation to CTCL lesions (lesion‐associated or independent), and (f) timing of pruritus in relation to the onset of visible lesions. Subpanels (g–i) show histograms representing the number of responses made in open, multiple‐answer questions regarding (g) the perceived character of the pruritus, (h) aggravating factors, and (i) alleviating factors.

All patients completed a CTCL Pruritus Questionnaire to assess major domains of pruritus (duration, frequency, course, localization, body area) as well as its relation to the disease and modulating factors (shown in Figure S1). Among the patient population suffering from pruritus related to CTCL (80.5%, *n* = 29/36), we observed a heterogeneous pattern of symptom‐specific features characterized in Figure 1. Pruritus was experienced daily (26.7%, *n* = 8/29), weekly (37.9%, *n* = 11/29) or in monthly intervals (26.7%, *n* = 8/29) (shown in Figure 1a). Symptoms were present for years (37.9%, *n* = 11/29) or more than 10 years (34.5%, *n* = 10/29) (shown in Figure 1b). Looking into the distribution pattern across the body, pruritus mostly appeared in multiple locations (58.6%, *n* = 17/29) (shown in Figure 1c) with a predominance of the lower extremities (28.6%, *n* = 12/29) (shown in Figure 1d). In relation to the clinical appearance of CTCL, pruritus occurred lesion‐associated (41.4%, *n* = 12/29) or independent of lesions (31.0%, *n* = 9/29) (shown in Figure 1e). In almost half of the patients, pruritus was felt before the lesion was visible (44.8%, *n* = 13/29) (shown in Figure 1f). Pruritus course varied from sudden attacks (82.8%, *n* = 24/29) to a constant course (16.7%, *n* = 4/24) (data not shown).

In summary, we observed a diverse range of pruritus characteristics among patients with CTCL. Pruritus commonly affected multiple body regions, often with a chronic and recurring course, and was frequently present for years or even decades. Notably, in many cases, symptoms preceded visible skin lesions and were not exclusively linked to lesional skin, highlighting the complex and heterogeneous nature of pruritus in CTCL.

To further characterize pruritus in CTCL, we assessed sensory qualities, triggers, and treatment responses through open, multiple‐answer questions. The character of the perceived sensation was most commonly described as itching (65.5%, *n* = 19/29), followed by superficial (27.6%, *n* = 8/29) and biting (27.6%, *n* = 8/29) (shown in Figure 1g). To better understand coping strategies present in our cohort, aggravating and alleviating factors were assessed. Stress (51.7%, *n* = 15/29), scratching (37.9%, *n* = 11/29) and sweat/exercise (31.0%, *n* = 9/29) were reported to worsen pruritic sensation (shown in Figure 1h) whereas cold temperature (31.0%, *n* = 9/29) was perceived as relieving (shown in Figure 1i). Successful pruritus alleviating therapy was reported in 58.6% (*n* = 17/29), most frequent with topical therapy including steroids and moisturizers (44.8%, *n* = 13/29) (shown in Figure S2a). 24.1% (*n* = 7/29) experience pruritus aggravation during therapy with nbUVB (5/29, 17.2%) or with topical treatment (2/29, 6.9%) (shown in Figure S2b).

Overall, patients described a range of sensory experiences and individualized responses to both external triggers and treatment interventions, underscoring the need for tailored pruritus management in CTCL.

### 3.3 Dermatology Quality of Life Index Strongly Correlates With a Three‐Item Pruritus‐Score

At the same time, the patients completed a questionnaire optimized for QoL evaluation in dermatological patients. Dermatology Quality of Life Index (DLQI) scores range from 0 to 30, with high values corresponding to a reduced QoL due to disease [8]. Across all patients, we saw a wide range corresponding to an absent to extremely large effect on patients' QoL (mean = 6.47, SD = 6.88, mean = 0, max =23) (shown in Table S1). DLQI was moderately associated with skin involvement (mSWAT) (*ρ* = 0.44, *p* < 0.01), but not disease stage (*ρ* = 0.08, *p* = 0.66) in patients with CTCL (shown in Table 2). Among patients reporting current or previous pruritus, pruritus‐specific characteristics such as duration (*ρ* = 0.11, *p* = 0.63), frequency (*ρ* = 0.31, *p* = 0.12), course (*ρ* = 0.36, *p* = 0.08) and localization (*ρ* = 0.36, *p* = 0.08) showed weak association to DLQI. Significant, moderate correlation was seen to worst experienced pruritus (*ρ* = 0.42, *p* = 0.04) and strong correlation to current perceived pruritus (*ρ* = 0.60, *p* < 0.01).

**TABLE 2:** Correlation of Dermatology Life Quality Index (DLQI) to CTCL clinical parameters and pruritus domains.

Item	*ρ*	adj.p
CTCL severity		
mSWAT	0.44	< 0.01**
Stage	0.08	0.66
Pruritus		
Duration	0.11	0.63
Frequency	0.31	0.12
Course	0.36	0.08
Localization	0.36	0.08
Worst experienced pruritus	0.42	0.04*
Current perceived pruritus	0.60	< 0.01**
Pruritus‐Score	0.71	< 0.01**

Abbreviations: CTCL, cutaneous T‐cell lymphoma; mSWAT, modified severity‐weighted assessment tool.

Significance of level *p < 0.05, **p < 0.01.

Based on these results, we selected skin involvement (mSWAT), current perceived pruritus, and worst experienced pruritus as relevant factors associated with the evaluated QoL. To develop a composite measure of disease burden, a Pruritus‐Score was constructed by combining those three components: current pruritus, worst pruritus, and mSWAT. As the variables differ in scale, with mSWAT ranging up to 400 and pruritus scores ranging from 0 to 10, we applied a scaling factor to mSWAT to ensure numerical comparability across components. Specifically, standard deviation (SD) was used as a balancing metric to align the relative contribution of each component to the total variance of the composite Pruritus‐Score. Scaling mSWAT by a factor of 10 reduced its SD from 22.56 to 2.26, matching current pruritus (SD = 2.24) and worst pruritus (SD = 2.26), in line with a comparable dispersion (shown in Figure S3a,b). This ensures equal influence on the variability of the composite score, while retaining interpretability. The internal consistency of the resulting Pruritus‐Score was assessed using Cronbach's alpha, indicating moderate internal consistency (*α* = 0.53, average inter‐item correlation = 0.27). This reflects the complementary contributions to Pruritus‐Score of subjective pruritus perception and objective skin involvement and supports its intended role as a clinically integrative composite measure. Overall, the Pruritus‐Score showed a strong correlation with Dermatology Life Quality Index (DLQI) scores (*ρ* = 0.71, *p* < 0.01), supporting its clinical and statistical relevance (shown Table 2).

### 3.4 Pruritus‐Score Is Associated With Impaired Cancer‐Specific Function Scales and Fatigue

To understand cancer‐specific aspects of QoL in CTCL, we investigated QoL evaluated with the QLQ‐C30 questionnaire [9]. QLQ‐C30 quantifies global health status and function scales starting from 100 (no impairment) decreasing to 0 (maximal impairment). Symptom scales are ranked with higher value (max = 100) corresponding to pronounced presence. Within our cohort, we observed a wide range of QoL considering global health status, function scales as well as symptom scales (shown in Table S1). To confirm the validity of the Pruritus‐Score, we correlated all QLQ‐C30 readouts as shown in Table 3. An increased Pruritus‐Score was significantly associated with impaired global health status (*ρ* = −0.64, *p* < 0.01). Further, Pruritus‐Score was moderate to strongly associated with physical (*ρ* = −0.44, *p* < 0.03), role (*ρ* = −0.53, *p* < 0.01), emotional (*ρ* = −0.57, *p* < 0.01) and social function (*ρ* = −0.60, *p* < 0.01). Assessment of cancer‐specific symptoms showed a strong correlation between Pruritus‐Score and fatigue (*ρ* = 0.50, *p* = 0.02).

**TABLE 3:** Correlation of Pruritus‐Score to outcome measures of QLQ‐C30.

QLQ‐C30 measure	*ρ*	adj.p
Global health status	−0.71	< 0.01**
Function scales		
Physical function	−0.44	< 0.03*
Role function	−0.53	< 0.01**
Emotional function	−0.57	< 0.01**
Cognitive function	−0.34	0.08
Social function	−0.60	< 0.01**
Symptom scales		
Fatigue	0.43	0.03*
Nausea and vomiting	0.30	0.16
Pain	0.36	0.07
Dyspnoe	0.28	0.18
Insomnia	0.26	0.21
Loss of appetite	0.12	0.56
Constipation	0.14	0.51
Diarrhoe	0.07	0.71
Financial difficulties	0.50	0.02*

Abbreviation: QLQ‐C30, EORTC Core Quality of Life Questionnaire.

Significance of level *p < 0.05, **p < 0.01.

## 4 Discussion

Pruritus is a debilitating symptom often associated with a negative impact on patients' QoL. Our study confirms previously published investigations, highlighting its relevance in CTCL [7, 17, 18, 19]. Still, pruritus is often neglected in clinical routine, potentially due to limited therapeutic options.

For the first time, we characterized detailed pruritus factors related to CTCL. In a small, representative cohort, we identified current pruritus, worst pruritus in the past, and disease severity as relevant factors linked to perceived QoL. The combination in the Pruritus‐Score is superior in identifying patients with impaired QoL than the individual parameters. It incorporates disease severity but also the accumulating consequences of chronic disease in a simple, fast applicable scoring system. This is in contrast to current pruritus questionnaires only focusing on pruritus‐specific aspects or QoL but lacking disease specificity [20, 21].

Only a minority of the patients included in the study receive successful pruritus‐specific therapy, highlighting the relevance of considering improving QoL by treating related symptoms with better therapeutic options. Pruritus perception varies between patients. Environmental and behavioral triggers such as scratching, stress, and elevated temperature frequently lead to aggravated pruritus. Manual violation of the superficial skin layer leads to a vicious worsening in an itch‐scratch cycle [22]. The constant desire to scratch is present in patients with pruritic skin and might not only worsen pruritus but also affect QoL. Improving the patients' awareness of their individual susceptibility might motivate them to initiate behavioral changes.

Further, we identified stress as a main factor associated with impaired pruritus. The influence of stress on pruritus might be mediated due to the hormonal landscape and subsequent modulation of the immune and nervous systems [23, 24]. Vice versa, chronic pruritus is related to increased stress, anxiety, and mood disorders [23]. The relation of stress and pruritus is a therapeutic target, especially in patients reporting chronic disease.

Focusing on cancer‐related symptoms assessed with QLQ‐C30, fatigue can occur as one of the consequences of disease severity as well as constant disturbance of pruritic skin. Chronic fatigue has been described in CTCL without correlation to pruritus [19]. As pruritus is frequently enhanced at night, sleeping disturbance can intensify fatigue [25]. Identifying present fatigue in a patient and raising the awareness can already have a profound impact on QoL. Adjustments in daily routine, psychoeducation up to pharmacological stimulative therapy can be considered.

## 5 Limitations

This is a single‐institutional study with a small study size, thus potentially limiting the generalizability of the results. Further, CTCL Pruritus Questionnaire was developed based on clinically relevant domains and informed by existing literature; it has not yet undergone formal psychometric validation. As such, Pruritus‐Score should be considered exploratory and be assessed in larger, international cohorts.

## 6 Conclusion

In conclusion, this study confirms the relevance of pruritus on QoL in patients with CTCL. The Pruritus‐Score offers clinicians a concise and disease‐specific tool to systematically assess pruritus in CTCL patients, integrating both subjective experience and objective disease severity. Its simplicity and strong correlation with QoL measures support its potential use in routine practice to identify patients at higher risk for impaired QoL who may benefit from supportive interventions. Beyond guiding clinical decision‐making, the score can serve as a monitoring tool to evaluate symptom progression or treatment response over time, thereby facilitating more personalized and proactive symptom management.

## Ethics Statement

This study protocol was reviewed and approved by the “Kantonale Ethikkommission Zurich” (BASEC 2023–00287) in compliance with the Helsinki Declaration. Written consent was acquired from all participants.

## Conflicts of Interest

Andrea Roggo and Yves Wüest do not have any conflicts of interest to disclose. Reinhard Dummer declares intermittent, project‐focused consulting and/or advisory relationships with Novartis, Merck Sharp & Dhome (MSD), Bristol‐Myers Squibb (BMS), Roche, Amgen, Takeda, Kyowa Kirin, Immunocore, Pierre Fabre, Sun Pharma, Sanofi, Catalym, Second Genome, Regeneron, Alligator, T3 Pharma, MaxiVAX SA, Pfizer, and touchIME outside the submitted work. Egle Ramelyte has served as an advisor and/or received speaking fees and/or travel support from Amgen, BMS, Eli Lilly, MSD, Novartis, Pfizer, Pierre Fabre, Roche, Sanofi, Galderma, and Philogen outside the submitted work.

## Supporting information


**Figure S1.** CTCL Pruritus Questionnaire. Overview of questions investigating pruritus in relation to CTCL. Evaluation strategy includes ranking of factors or qualitative evaluation.
**Figure S2.** Therapy alleviating or aggravating perceived pruritus. Histogram showing therapeutic approaches leading to (a) improved or (b) worse pruritus. Number of responses in multi‐answer questions are indicated. Abbreviations: ECP (extracorporeal phrotopheresis), nbUVB (narrowband ultraviolet phototherapy).
**Figure S3.** Characteristics of individual factors contributing to Pruritus‐Score. (a) Visualization of the relationship between mSWAT scaling by a factor ranging from 1 to 40 and resulting standard deviation. (b) Histogram showing distribution of scaled mSWAT (mSWAT/10), worst experienced pruritus (Worst Pruritus) and current perceived pruritus (Current Pruritus) across all patients. Abbreviations: mSWAT (modified severity‐weighted assessment tool).

**Table S1.** Quality of Life readouts across all patients. Overview of descriptive statistics for the two QoL questionnaires DLQI and QLQ‐C30 across the complete investigational cohort. Abbreviations: DLQI (Dermatology Quality of Life Index), SD (Standard deviation), QLQ‐C30 (EORTC Core Quality of Life Questionnaire).

## Data Availability

The data that support the findings of this study are not publicly available due to privacy reasons but are available from the corresponding author (E.R.) upon reasonable request.
