# Supplementary material for: Pruritus Is an Indicator for Quality of Life in Cutaneous T‐Cell Lymphoma
Source: J Dermatol. 2025 Jul 18;52(9):1404–10. doi: 10.1111/1346-8138.17847 (PMC12411805; doi:10.1111/1346-8138.17847)

SUPPLEMENTARY FIGURES

Fig. S1. CTCL Pruritus Questionnaire

Item	Values	Evaluation and ranking strategy
Duration	Days<br>A few weeks<br>Months<br>Years<br>>10 years<br>Ø not specified	1<br>2<br>3<br>4<br>5<br>2.5
Frequency	Multiple times per month<br>Multiple times per week<br>Daily<br>Ø not specified	1<br>2<br>3<br>1.5
Course	Sudden, intermittent<br>Constant<br>Ø not specified	1<br>2<br>1
Localization	Single location<br>Multiple locations<br>Entire body<br>Ø not specified	1<br>2<br>3<br>1.5
Body area	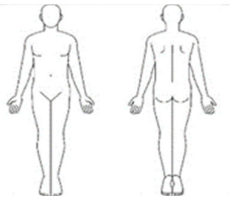 Head/Neck<br>Upper extremity<br>Trunk<br>Lower extremity	Qualitative evaluation of marked region
Lesion dependency	Lesion associated<br>Not limited to lesion<br>Independent	Qualitative evaluation
Timing	Before lesion<br>Simultaneous with lesion<br>After lesion	Qualitative evaluation
Pruritic sensation	Character of pruritus, free answers	Qualitative evaluation
Improving factors	Environmental and behavioral triggers (cold, warmth), treatment, free answers possible	Qualitative evaluation
Aggravating factors	Soft factors (stress, relaxation, warmth, exercise, touch, pressure), treatment, free answers possible	Qualitative evaluation
Pruritus-Score	Current pruritus<br>Worst pruritus experienced<br>mSWAT/10	(1-10)<br>(1-10)<br>(1-40)
		Sum: /60

Fig. S2. Therapy alleviating or aggravating perceived pruritus

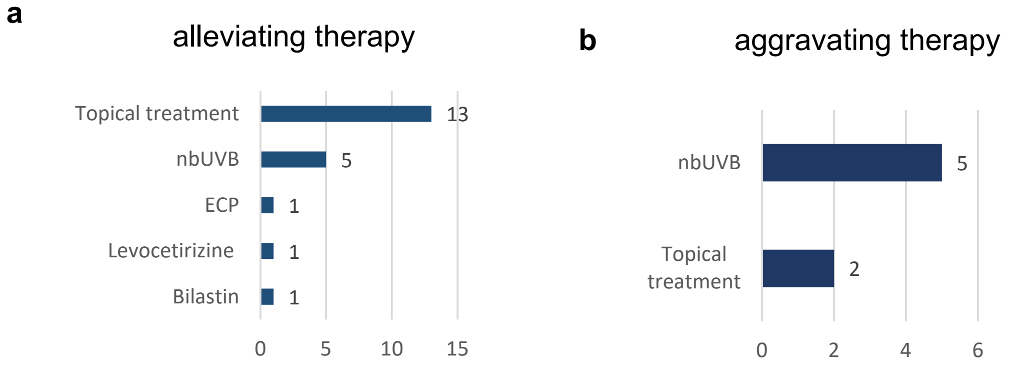

Fig. S3. Characterization of individual factors contributing to Pruritus-Score

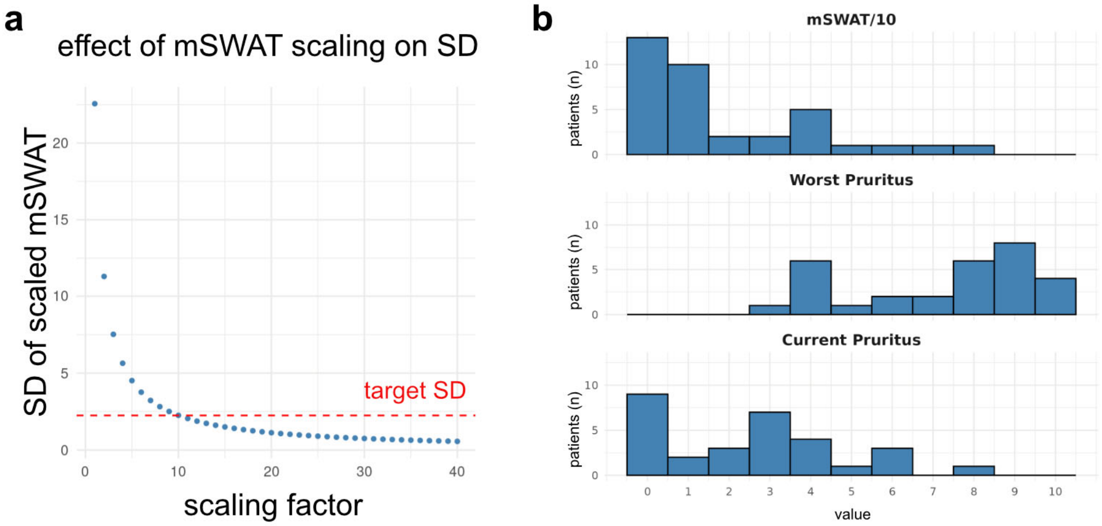

Supplement: Supplementary file 1 — Figure S1. CTCL Pruritus Questionnaire. Overview of questions investigating pruritus in relation to CTCL. Evaluation strategy includes ranking of factors or qualitative evaluation. Figure S2. Therapy alleviating or aggravating perceived pruritus. Histogram showing therapeutic approaches leading to (a) improved or (b) worse pruritus. Number of responses in multi‐answer questions are indicated. Abbreviations: ECP (extracorporeal phrotopheresis), nbUVB (narrowband ultraviolet phototherapy). Figure S3. Characteristics of individual factors contributing to Pruritus‐Score. (a) Visualization of the relationship between mSWAT scaling by a factor ranging from 1 to 40 and resulting standard deviation. (b) Histogram showing distribution of scaled mSWAT (mSWAT/10), worst experienced pruritus (Worst Pruritus) and current perceived pruritus (Current Pruritus) across all patients. Abbreviations: mSWAT (modified severity‐weighted assessment tool). [file JDE-52-1404-s002.pdf]
