# Supplementary material for: Pruritus Is an Indicator for Quality of Life in Cutaneous T‐Cell Lymphoma
Source: J Dermatol. 2025 Jul 18;52(9):1404–10. doi: 10.1111/1346-8138.17847 (PMC12411805; doi:10.1111/1346-8138.17847)
Supplement: Supplementary file 2 — Table S1. Quality of Life readouts across all patients. Overview of descriptive statistics for the two QoL questionnaires DLQI and QLQ‐C30 across the complete investigational cohort. Abbreviations: DLQI (Dermatology Quality of Life Index), SD (Standard deviation), QLQ‐C30 (EORTC Core Quality of Life Questionnaire). [file JDE-52-1404-s001.docx]

# SUPPLEMENTARY TABLE

# Tab. S1. QoL readouts across all patients

| CTCL cohort (n=36) | | | | |
| --- | --- | --- | --- | --- |
|  | Mean | Min | Max | SD |
| **DLQI** | 6.47 | 0 | 23 | 6.88 |
| **QLQ-C30**  **Global health status** | 68.33 | 33.33 | 100.00 | 18.23 |
| **Function scales** |  |  |  |  |
| Physical function | 78.89 | 26.67 | 100.00 | 27.52 |
| Role function | 72.78 | 0 | 100.00 | 35.15 |
| Emotional function | 66.94 | 0 | 100.00 | 28.15 |
| Cognitive function | 80.56 | 0 | 100.00 | 24.79 |
| Social function | 73.33 | 0 | 100.00 | 32.64 |
| **Symptom scales** |  |  |  |  |
| Fatigue | 38.15 | 0 | 100.00 | 37.45 |
| Nausea and vomiting | 7.78 | 0 | 100.00 | 19.93 |
| Pain | 22.78 | 0 | 100.00 | 35.42 |
| Dyspnoe | 14.44 | 0 | 100.00 | 25.80 |
| Insomnia | 28.89 | 0 | 100.00 | 32.44 |
| Loss of appetite | 17.78 | 0 | 100.00 | 32.44 |
| Constipation | 22.22 | 0 | 100.00 | 35.58 |
| Diarrhoe | 8.89 | 0 | 66.67 | 19.44 |
| Financial difficulties | 17.78 | 0 | 100.00 | 32.44 |

DLQI (Dermatology Quality of Life Index), SD (Standard deviation), QLQ-C30 (EORTC Core Quality of Life Questionnaire)
